# Development of a robust and quantitative high-throughput screening method for assessing phenotypic variation in large *Neisseria meningitidis* isolate collections

**DOI:** 10.1016/j.mex.2023.102091

**Published:** 2023-03-02

**Authors:** Robeena Farzand, Megan De Ste Croix, Neelam Dave, Christopher D. Bayliss

**Affiliations:** a Department of Genetics and Genome Biology, University of Leicester, United Kingdom; b Academic Quality and Development, Staffordshire University, United Kingdom

**Keywords:** High-throughput, Phenotypic screening, *Neisseria meningitidis*, Multi-assay stock, Meningococcus, High-throughput phenotypic screening method for assessing phenotypic variation

## Abstract

Genome-wide association studies are a powerful approach for identifying determinants of disease. For infectious diseases, high throughput assays are required for measuring the variance in multiple virulence-related phenotypes of large bacterial isolate collections and for association of this phenotypic variance with genotype. The primary limiting factors are cost, effectiveness and a standardized inoculum. A method was developed to create an inoculum array of multiple isolates that could be used for a series of high-throughput multi-isolate phenotypic investigations in a laboratory setting. A key starting point was the standardisation of the inoculum by production of identical batches of each isolate from cells grown to mid-log phase. Cultures with pre-determined optical densities were aliquoted in set patterns into multiple multi-well plates containing 50% glycerol and stored at -80 °C. Prior to a specific assay, an inoculum plate was defrosted and subjected to a brief period of incubation. Control strains can be placed on each plate in order to control for intra-assay variability. A high throughput screen is described in detail for quantification of biofilm formation. This example utilised the crystal violet staining method and multi-assay stock plates containing 16 meningococcal isolates.

• Multi-assay stock plate of exponentially growing isolates is cost-effective and simple to implement in a laboratory setting.
• This method would predict realistic standard deviations for multiple isolates in phenotypic assays and generate data for performance of power calculations for genotyping.
• This method has the potential to identify both known and unknown genetic determinants of phenotypic variability for each tested isolate when paired with genetic analysis of whole genome sequencing data.

## Graphical abstract

**Figure:**
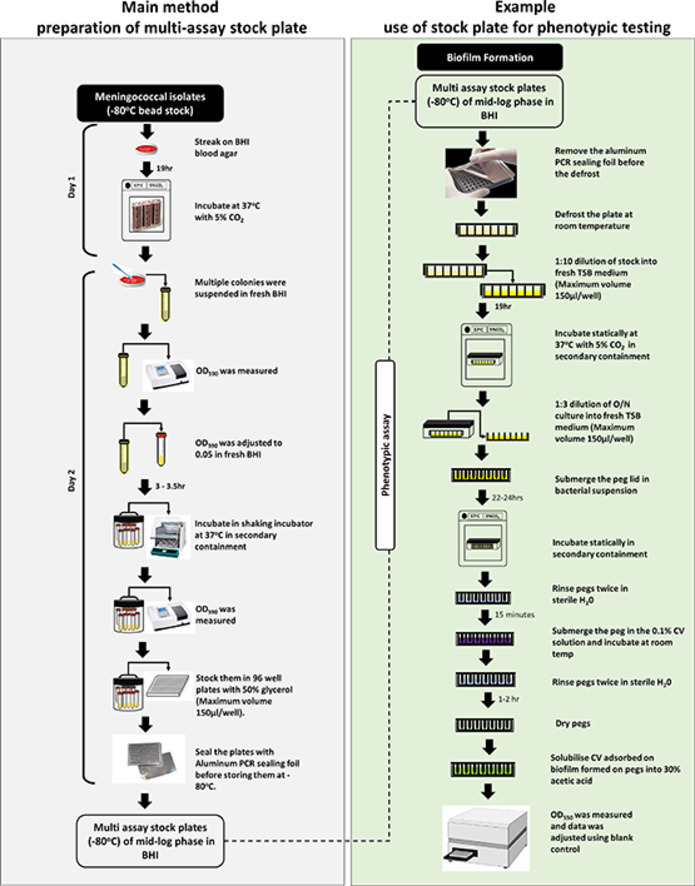

Specifications table

**Table:** 

Subject area:	Immunology and Microbiology
More specific subject area:	*Microbiology*
Name of your method:	High-throughput phenotypic screening method for assessing phenotypic variation
Name and reference of original method:	*Not applicable*
Resource availability:	*Details are provided in method section.*

## Background

Meningococcal infection is a worldwide issue that manifests as sporadic, hyper-sporadic, and epidemic disease [1]. There are an estimated 1.2 million cases of meningococcal infection each year, with a death toll of -135,000 worldwide [2]. Disease patterns differ greatly over time and across geographical areas, age groups, and bacterial serogroups. Most of the disease is caused by a few genetically defined *N. meningitidis* lineages (clonal complexes) that can emerge and spread globally [3]. Genome-wide association studies are a powerful approach to identification of disease determinants [4]. These studies require high-throughput analysis of multiple isolates of specific lineages in order to determine the extent of phenotypic variation and to associate this variation with specific genetic variants.

**Fig. 1:**
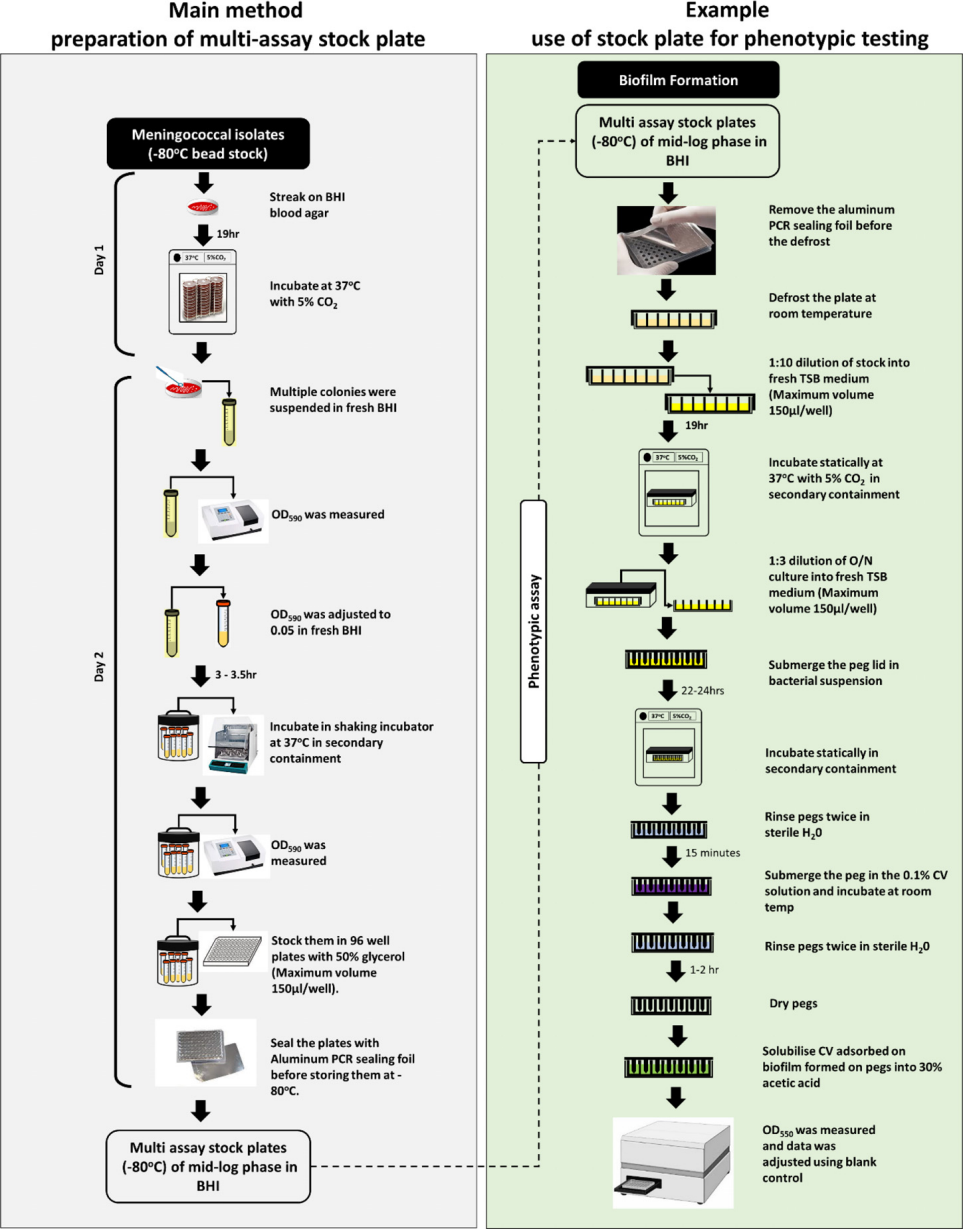
Overview of method for preparation of multi-assay stock plates and high throughput phenotypic testing. Two flow diagrams are shown for high-throughput phenotypic screening of multiple meningococcal isolates. The left (grey) panel depicts a standardised stocking method for preparation of multiple assay stock plates for 96 isolates. Note that this method is adaptable to any bacterial species that can be grown in broth culture. The right (green) panel depicts an example of high throughput phenotypic testing of biofilm formation utilising a multi assay stock plate produced using our standardised stocking method. Note that this format is adaptable to other phenotypic screens.

A key starting point for high throughput screening assays is to develop a technique for producing an inoculum array of large numbers of isolates that can be used for several high-throughput multi-isolate phenotypic studies. The technique needs to be affordable and simple to implement in a laboratory setting. The method should be able to predict realistic standard deviations for multiple isolates in phenotypic assays and generate data for performance of power calculations for genotyping. When combined with genetic analysis of whole genome sequence data, or other types of genetic variation, for each assayed isolate, this approach has the potential to detect both known and novel genetic determinants of phenotypic variability. This will include exploration of whether single-nucleotide variants are associated with significant differences in a specific phenotype. Fig. 1 depicts an overview of our method, which involves growing multiple meningococcal isolates to a specific point in the logarithmic phase of growth and then archiving these isolates in 96 well plates at -80 °C. These stock plates can then be utilised as input to any method that is adaptable to a multi-well high throughput format. In on-going work, six distinct phenotypic assays have been adapted, validated and verified for analyses of multiple isolates using our high throughput approach (Farzand and Bayliss, unpublished data). These six tests evaluate biofilm formation, sensitivity to serum, adherence to A549 (lung carcinoma epithelial) cells, and growth in both minimal and enriched media. Assays have been performed on ∼300 MenW cc11 and ∼200 MenY cc23 isolates with equal proportions of disease and carriage isolates. Here, we addressed use of these stock plates for one of these phenotypic investigations and show the quantification of biofilm formation for 16 isolates as an example.

## Method details

### General safety aspects

*Neisseria meningitidis* is listed in the UK as a containment level (CL) 2+ organism [5] and all work performed herein was undertaken in a dedicated CL2+ facility. Methods were subject to risk assessments that aimed to minimise the potential for aerosol production, contamination of equipment or spillages. Work with open cultures or plates of *N. meningitidis* were performed in a microbiological safety cabinet (MSC). For the biofilm assay, *N. meningitidis* inactivation by treatment with 30% acetic acid for one minute or more was confirmed as part of the development of a protocol. The protocol development also aimed to minimise the quantity and length of time that containers of 'live' *N. meningitidis* were open. All equipment and workspaces were sterilised with 70% IMS (Industrial Methylated Spirit) before and after each experiment.

## Method

### Preparation of multiple assay stock

Multi assay stock plates of large numbers of isolates were prepared in batches to ensure reproducibility and minimise the potential for genetic variation (*e.g.* phase variation) to impact replicate results of a specific strain during phenotypic analysis. The quantities described herein are sufficient for preparation of fifteen 96-well plates.

### Day-1

(1) A glycerol storage tube for a meningococcal isolate is transferred from the -80 °C freezer to the MSC within the CL2+ facility.
(2) The culture is opened, and an aliquot is scrapped from the surface using a plastic loop. Care must be taken not to use excessive force to avoid breakage of the loop or displacement of material out of the tube. These precautions will reduce the chances of a minor spill incident.
(3) Strains are plated on to bovine heart infusion agar (BHIA) supplemented with 5% horse blood (Sigma Aldrich, UK). The plates are placed upside-down in agar plate racks and incubated at 37 °C with 5% CO2 for -18 h.

### Day-2

(4) Using a sterile disposable loop, a sweep of colonies from the overnight plate growth are suspended into 2 mL BHI broth in a 15 mL falcon and the optical density (OD) of this culture is measured at 590 nm.
(5) An aliquot of this initial culture was utilised to prepare a fresh BHI culture (2 mL) with a final OD_590_ of 0.05. This new culture was incubated in secondary containment for 3 h at 37 °C with shaking to allow bacterial replication to enter into the exponential growth phase and to reach an OD_590_ of 0.3–0.4.
(6) During the bacterial replication phase, 75 µL of a 50% (volume/volume) glycerol stock is added to each well of a polypropylene 96 well plate. Polypropylene plates are used due to having high durability at very low temperatures.
(7) Once the bacterial culture has attained the correct OD_590_, 75 µL of bacterial culture is transferred to a specific well of the 96 well plate as determined by a pre-defined plate map.
(8) The plates are sealed using an adhesive foil PCR seal ensuring that there is good contact between the seal and the plate. Replace the lid of the plate over the foil seal.
(9) Label the plates and store in the -80 °C freezer.
(10) Extra strains can be added to plates on subsequent days. In this case, plates are placed on dry ice to ensure that the previously frozen stocks do not defrost.

### Use of multi-assay stock plates for a phenotypic analysis of biofilm formation

Multi-assay stock plates are for single use due to freeze thawing of bacterial cultures resulting in loss of cell viability.

### Step-1 “Preparation for assay”

Steps 1 to 4 are common to all phenotypic investigations. Here, quantification of biofilm formation is explained as one example of a high throughput phenotypic investigation in a standard CL2+ facility.

(1) 96-well multi assay stock plate is removed from the -80 °C freezer and placed into the MSC. Removal of the PCR foil MUST be completed before the plate is fully defrosted in order to avoid spillages. An alternative method is to sterilize the PCR foil by wiping with 70% IMS and then pierce the foil with sterile pipette tips. After either method, plates are left at room temperature for a significant period of time to allow for complete defrosting of the bacterial glycerol suspensions.
(2) Flat-bottomed 96-well plates are prepared by adding 135 µL Tryptic Soy Broth (TSB) (Oxoid, UK) and 15 µL of defrosted culture. Note that any bacterial growth medium could be used at this point as required for a specific phenotypic screening method.
(3) Plate lids are securely attached with autoclave tape before being securely placed in secondary containment.
(4) Plates are incubated statically at 37 °C with 5% CO_2_ overnight (18–19 h).

### Step-2 “Growth and staining of biofilms on pegs”

(5) Aliquots of overnight bacterial cultures are transferred into a fresh 96 well plate with addition of 50uL of culture to 100uL BHI. The culture density is measured at OD_590_ using a plate reader.
(6) Peg lids are inserted, and plates are incubated statically in secondary containment at 37 °C with 5% CO_2_ for 22–24 h. Note: the method explained by Harrison et al. (2010) [6] was modified for the growth of meningococcal strains on pegs.
(7) After incubation, the peg lids are washed twice by gently submerging them in a 96 well plate containing 200µL sterile ddH_2_0 per well.
(8) The biofilms formed on pegs are stained by transferring pegs to a 96 well plate containing 125µL of 0.1% crystal violet (CV) solution per well.
(9) The pegs are submerged in the crystal violet solution at room temp for a minimum of 15 min.
(10) After staining, the pegs are rinsed twice by gently submerging them in a 96 well plate containing 200 µL sterile ddH_2_0 per well.
(11) The pegs are dried at room temperature within the MSC for at least 1 h up to overnight.

### Step-3 “Quantification of biofilm”

(12) To solubilize the CV, the peg lid is transferred to a new 96 well plate containing 125 µL of 30% acetic acid (in water). This is followed by incubation at room temperature for a minimum of 15 mins. (Note: 100% killing of >10^10^ *N. meningitidis* was observed to occur within 15 min of incubation in 30% acetic acid; killing was assessed as part of the risk assessment for this protocol and indicates that these plates can be removed from the CL2+ facility from this step onward; similar approaches should be followed during development of protocols for other phenotypes).
(13) Biofilm formation, as determined by CV staining, is quantified using a plate reader at 550 nm. A control well containing only 30% acetic acid is used as the blank for correction of the final readings.
(14) For comparative assessments of multiple isolates, CV readings are normalised to a known biofilm producer or positive control.

## Method validation

### Test sample of multi-assay stock plates

To validate the multi-assay stock plate format, multiple plates were prepared for 16 MenW cc-11 isolates (8 invasive, 8 carriage). These plates were utilised for testing recovery of bacterial growth, viability, and phenotypic data.

### Viability of multi-assay stock plate

**Fig. 2:**
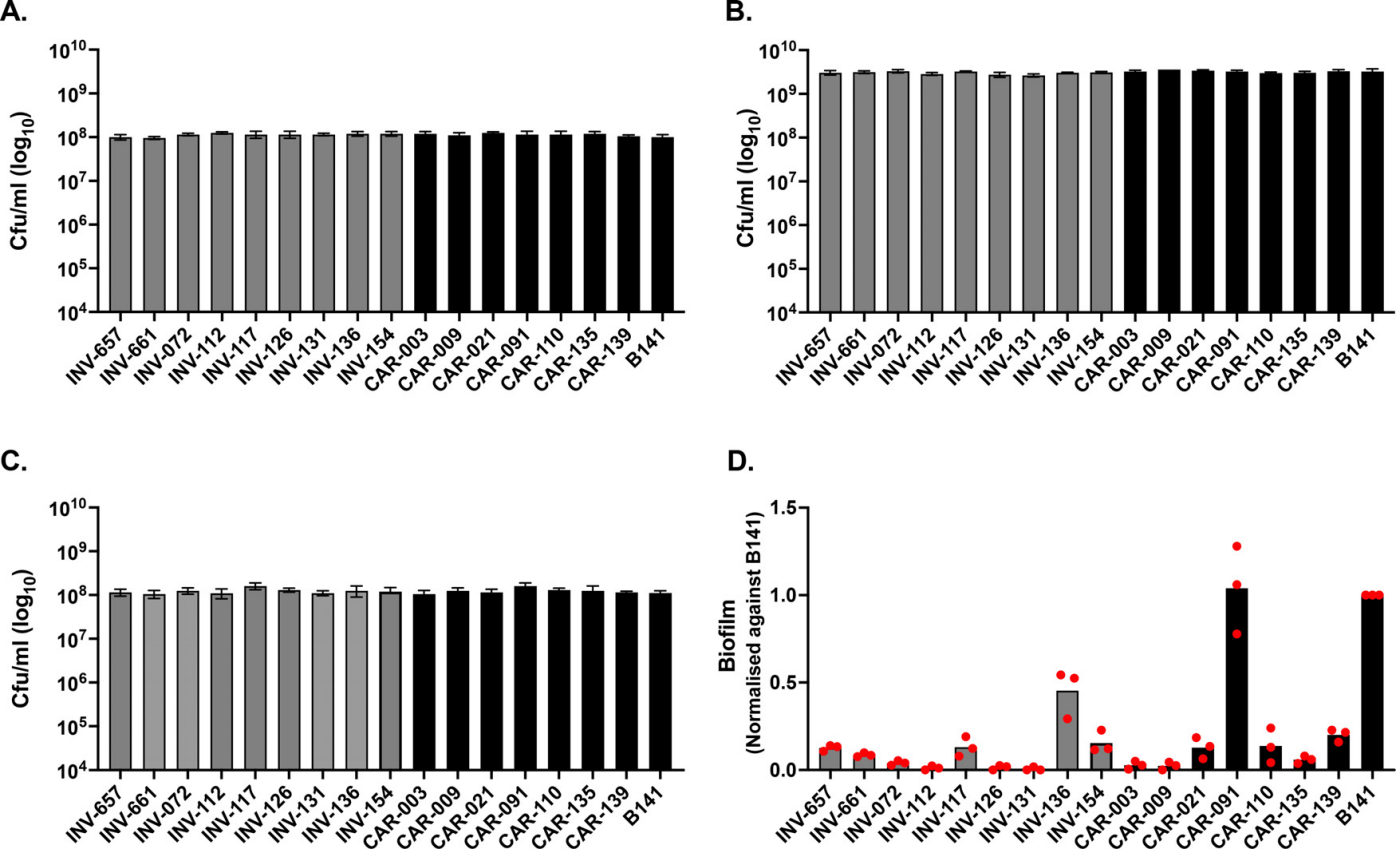
Validation of the method developed for *N. meningitidis* high-throughput phenotypic screening. (a) Viability validation test for recovery after freezing for a multi-assay stock plate. Viability was examined by performing serial dilutions directly of aliquots from each well following multi-assay stock plate defrosting. A spot plate method was employed to determine the CFU counts. (b) Viability validation test for overnight growth. The stock was defrosted and then utilised to inoculate a new plate containing BHI broth with a 1:10 dilution ratio. This plate was incubated overnight at 37 °C with 5% CO2 before the CFU/mL was quantified using the spot plate method. (c) Viability validation test for exponential growth. Plates were manipulated as for B except that CFU/mL were assessed after three hours of growth. D) Validation of high throughput phenotypic data collection. Quantification of the biofilms formed by multiple isolates was assessed using the CV staining method. Observed OD550 measurements were normalised against the values obtained for a high biofilm forming isolate, B141. Bars represent averages of three biological replicates. Red dots represent average values of three technical replicates obtained from three independent experiments. Variability in each of the different steps (panel a–c) is numerically quantified and listed in Table 1.

Long-term bacterial isolate storage is best accomplished through freezing or lyophilisation (freeze-drying). For frequently recovered isolates, freezing is the most convenient storage method because the cultures do not need to thaw completely each time they are removed from frozen storage. However, freeze thawing reduces cell viability; to mitigate this effect, multi-assay stock plates are prepared in batches for single use. To test if defrosting plates affects viability, a colony forming unit (CFU) test was undertaken to determine the numbers of viable cells. The CFU of randomly selected isolates from the stock plates are shown in Fig. 2a. No significant differences were detected between the CFU of the various isolates indicating that viability had been maintained and that cultures were comparable.

### Validation of reproducibility of multiple isolates

To ensure that isolates stocked on multi-assay plates exhibit similar levels of re-growth prior to performance of phenotypic screening, the stock plate cultures were subject to a 1:10 dilution into fresh media in another 96-well flat bottom plate and allowed to grow for 18 hr. Determination of viable cells in a CFU assay indicated that all isolates had been recovered at similar levels and hence were viable for growth in culture media (Fig. 2b).

The final stage of preparing the multiple isolates for high throughput phenotypic screening was also assessed. The overnight cultures were subcultured (1:10) into a new 96 well plate containing BHI and incubated until mid-exponential growth phase (-3h). The OD_590_ was measured before and after growth using a multi-well plate reader, and the CFU count was quantified. Results in Fig. 2c reveal comparable numbers of viable bacteria that can be employed with confidence in subsequent investigations. (Tables 2 and 3)

**Table 1:** Quantification of the variability in different steps of multi assay stock plate preparation.

Isolates	Frozen (panel A of Fig. 2)			O/N culture (panel B of Fig. 2)			Exp. growth (panel C of Fig. 2)		
	Mean CFU	STDEV^a^	CV^b^	Mean CFU	STDEV	CV	Mean CFU	STDEV	CV
INV-657	1.00E+08	1.41E+07	0.14	3.05E+09	3.54E+08	0.12	1.15E+08	2.12E+07	0.18
INV-661	9.50E+07	7.07E+06	0.07	3.15E+09	2.12E+08	0.07	1.05E+08	2.12E+07	0.20
INV-072	1.15E+08	7.07E+06	0.06	3.30E+09	2.83E+08	0.09	1.25E+08	2.12E+07	0.17
INV-112	1.25E+08	7.07E+06	0.06	2.85E+09	2.12E+08	0.07	1.10E+08	2.83E+07	0.26
INV-117	1.15E+08	2.12E+07	0.18	3.25E+09	7.07E+07	0.02	1.60E+08	2.83E+07	0.18
INV-126	1.15E+08	2.12E+07	0.18	2.75E+09	3.54E+08	0.13	1.30E+08	1.41E+07	0.11
INV-131	1.15E+08	7.07E+06	0.06	2.65E+09	2.12E+08	0.08	1.10E+08	1.41E+07	0.13
INV-136	1.20E+08	1.41E+07	0.12	3.05E+09	7.07E+07	0.02	1.25E+08	3.54E+07	0.28
INV-154	1.20E+08	1.41E+07	0.12	3.10E+09	1.41E+08	0.05	1.20E+08	2.83E+07	0.24
CAR-003	1.20E+08	1.41E+07	0.12	3.25E+09	2.12E+08	0.07	1.05E+08	2.12E+07	0.20
CAR-009	1.10E+08	1.41E+07	0.13	3.60E+09	2.12E+08	0.06	1.25E+08	2.12E+07	0.17
CAR-021	1.25E+08	7.07E+06	0.06	3.40E+09	1.41E+08	0.04	1.15E+08	2.12E+07	0.18
CAR-091	1.15E+08	2.12E+07	0.18	3.25E+09	2.12E+08	0.07	1.60E+08	2.83E+07	0.18
CAR-110	1.15E+08	2.12E+07	0.18	3.00E+09	1.41E+08	0.05	1.30E+08	1.41E+07	0.11
CAR-135	1.20E+08	1.41E+07	0.12	3.05E+09	2.12E+08	0.07	1.25E+08	3.54E+07	0.28
CAR-139	1.05E+08	7.07E+06	0.07	3.30E+09	2.83E+08	0.09	1.15E+08	7.07E+06	0.06
B141	1.00E+08	1.41E+07	0.14	3.25E+09	4.95E+08	0.15	1.10E+08	1.41E+07	0.13

a Standard deviation

b Co-efficient of variation

**Table 2:** Cost associated with primary reagents for one iteration of the main multi assay stock plate method^1^.

Main Method			
Reagent	Brand/Source	Quantity	Cost (£)
BHIA	Oxoid	500 gm	126.65
BHI	Oxoid	500 gm	78.97
Chlorine Bleach Sanitiser Disinfectant Tablets	Titan	600 Tabs/ pack	65.30
Foil PCR seal	4titude	100 unit	125.05
Corning round bottom polypropylene 96 well plate (For multi-assay stocks)	Corning	100 unit	264.44
Petri dishes (cat. no. 08–757–12)	Thermo Fisher Scientific	100 units	36.11
15-mL falcon tubes	Thermo Fisher Scientific	500 units	72.38

1 These reagents are sufficient to produce more than 100 stock plates. Note; petri dishes, falcon tubes and 96-well plates are subject to number of isolates used for stocking.

**Table 3:** Cost of reagents for high throughput biofilm assay using multi assay stock plates as inoculum^1^.

Phenotypic assay (Biofilm formation)			
Reagent	Brand/Source	Quantity	Cost (£)
TSB	Liofilchem	500 gm	77.40
Crystal Violet stain	Sigma-Aldrich	100 gm	162.45
Acetic acid (100%)	Fisher Chemicals	1.0 Litre	105.84
Peg lids (Nunc Immuno TSP, cat. no. 445,497)	Thermo Fisher Scientific	50 units	291
Microtiter-plates with lids (cat. no. 167,008)	Thermo Fisher Scientific	50 units	58.56

1 These reagents are sufficient for assaying more than 100 plates with 3 experimental and technical replicates: Note; peg lids and microtiter plates are subject to number of strains and experimental tests.

### Validation of the generation of high throughput phenotypic data

To test if phenotypic data could be produced in a high throughput manner, the test plate was subjected to quantification of biofilm formation. Fig. 2d displays measurements of biofilm development and shows data obtained from three biological replicates, our results shows that data was reproducible between replicate experiments.

### Reagents and materials

○ BHIA (OXOID)
○ BHI (OXOID)
○ TSB (LIOFILCHEM)
○ Glycerol >= 99% (Fisher Chemicals)
○ 70% IMS
○ Chlorine Bleach Sanitiser Disinfectant Tablets (TITAN). Note: Concentrated bleach was used for disinfection of liquid *N. meningitidis* waste
○ 0.1% Crystal violet (prepared in ddH_2_0 and filtered using a 0.45 nm filter to remove any residual particles)
○ 30% acetic acid (prepared in ddH_2_O)
○ Autoclave (Certo Clav Classic)
○ Static Incubator (with 5% CO_2_; PHCBI)
○ Shaking Incubator (Innova 4000)
○ Secondary containment for multi-well plates (clipped lid boxes)
○ Secondary containment for 15 mL tubes for shaking incubator
○ Secondary containment for transferring of meningococcal isolates to and from freezer to MSC in CL2+ lab (clipped lid boxes)
○ MSC Class I
○ MSC Class II
○ Microtiter plate reader (Molecular Devices)
○ Portable Cell Density meter (Biochrom Ultrospec 10)
○ Balance (Denver instrument)
○ Single channel pipette (20–200 µL)
○ Single channel pipette (100–1000 µL)
○ Pipette (12 channel, 30–300 µL)
○ Pipette tips (20 µL, 200 µL, and 1000 µL)
○ Inoculation loop
○ Scissors
○ Foil PCR seal
○ Autoclave tap
○ Parafilm (to seal the agar, multi-well plates before disposal)
○ Plate sealers (autoclave tape)
○ Corning round bottom polypropylene 96 well plate (For multi-assay stocks)
○ Peg lids (Nunc Immuno-TSP, Nunc, cat. no. 445,497) Note; The Nunc Immuno-TSP lids can be used to grow biofilms in addition to being supports for solid-surface enzyme-linked immunosorbent tests. These lids lack the plastic backing and break points that make it easier for pegs to come off and have a polystyrene surface that has been chemically changed. They also have an overall positive electrostatic charge. The inoculum reservoir trough that comes with these lids can be switched out for a microtiter plate at the user's option.
○ Microtiter plates with lids (96-well; Nunc, cat. no. 167,008) Note; for best results, we recommend the use of clear, flat-bottomed, sterile Nunclon microwell plates that are packaged with lids.
○ Petri dishes (Fisherbrand, cat. no. 08–757–12)
○ 15 mL falcon tubes
○ Pipette dispenser
○ Pipettes (10 mL; Corning Costar, cat. no. 4012)
○ Pipettes (25 mL; Corning Costar, cat. no. 4489)
○ Paper towel

## Ethics statements

Not applicable

## CRediT authorship contribution statement

**Robeena Farzand:** Conceptualization, Methodology, Validation, Data curation, Writing – original draft. **Megan De Ste Croix:** Conceptualization, Methodology, Validation. **Neelam Dave:** Methodology, Writing – review & editing. **Christopher D. Bayliss:** Supervision, Conceptualization, Methodology, Writing – review & editing.

## Declaration of Competing Interest

The authors declare that they have no known competing financial interests or personal relationships that could have appeared to influence the work reported in this paper.

## Data Availability

Authors confirms that data presented in this article is permitted to be share with reader.
